# Who Is the “Selfish Demagogue?”

**Published:** 1861-02

**Authors:** 


### EDITORIAL AND MISCELLANEOUS.

### Who is the "Selfish Demagogue?"

As we expected, the New York Medical and Surgical Reporter is exercised considerably on account of the views expressed in the January number of our Journal, touching the American Medical Association, secession &c. Those views, if carried out, would not advance the special *interest* sought to be promoted by the Reporter. Neither would they agree very well with the deceptive system of government attempted to be enforced upon medical institutions of learning, by the American Medical Association, and supported so zealously by this New York Weekly Medical Journal. The editors of this valuable medical Weekly, as forcible, correct and truely scientific writers, are not surpassed by any; and their weekly periodical, laiden with practical and theoretical truths, is welcomed as one of our very best exchanges. Occasionally, however, we are pained to see the stream of science made turbid by the attempt to connect the government of local institutions with the duties of a national body, organized for the advancement of science by yearly reunions and interchange of opinions on purely scientific subjects.—This doctrine we think has a tendency to make *science subservient to selfish interests*, and yet the Recorder is indignant when it is condemned.

In proof of this we refer the reader to the onslaught made by the Reporter upon the New Orleans Gazette for opinions adverse to the Association and its recent action. Again, in reply to our editorial for January, in which we express the opinion that certain resolutions passed by that body were deceptive, that the real object was to effect selfish ends &c., the Reporter says, "in spite of the Atlanta Journal the day will never come when science will be made subservient to the interests of selfish demagogues." Now, what are the facts? The association, which assembled in Connecticut in June last, and composed principally of Northern delegates, passed, among other things, a resolution requiring students to make a certain interval between their courses of lectures, which virtually does, and was intended to cut off patronage from certain colleges in which the majority of the Association did not feel an *interest.* This was done with the ostensible object of improving the system of Medical education, but with the real view of stricking a blow at particular institutions. This looks very much to us like making a body, organized for the advancement of science, "subservient to the interests of selfish demagogues;" and the advocate of such hypocritical maneuvering and wire-working looks to us like a veritable demagogue.

So far form wishing to make science sectional, or to prostitute the great cause to the low, selfish wire-working of mere tricksters we are for making Medical Science universal, by separating it from sectional and institutional quarrels, and raising it above the polution of the selfish demagogue, who would cloak his ugly form with her pure rube to strike a dagger to the heart of a rival institution. Let the government of the profession, and of medical institutions be made with reference to the political government and other circumstances surrounding a particular people; and let bodies organized for the advancement of science adhere to the object of their organization, whether north or South, in

Europe or America. Then it would be that contributors from the East and from the West, from the North and from the South, could meet upon a common level for the advancement of the great cause. Call not the sectional favors granted by a medical or political congress the legitimate operations of scientific bodies and republican governments. In both, we say, if legitimate action cannot be preserved, and the object and compact cannot be maintained, on account of overgrown sectional majorities, let *secession* come, and the people of each section govern themselves in a manner suited to the circumstances which surround them. In this way science may be kept aloof from governmental strife. But to talk of governing the actions of men toward each other, whether medical or otherwise, beyond the boundary of political govenment, is, to say the least of it, very ridiculous. In conclusion, we would say to the Reporter, and to all others with similar views, that if they indulge the hope that Georgia will ever again be represented, either in the medical or political congress of the late United States, or that this noble State or her people will ever bite the dust and ask the forgiveness of their oppressors "they are reckoning without their hosts."

